# Effect of acupuncture treatment on post-stroke cognitive impairment: A randomized controlled trial

**DOI:** 10.1097/MD.0000000000023803

**Published:** 2020-12-18

**Authors:** Yuzheng Du, Lili Zhang, Wei Liu, Chang Rao, Boxuan Li, Xi Nan, Zefang Li, Hailun Jiang

**Affiliations:** a First Teaching Hospital of Tianjin University of Traditional Chinese Medicine; b National Clinical Research Center for Chinese Medicine Acupuncture and Moxibustion, Tianjin, China.

**Keywords:** acupuncture, cognitive impairment, randomized controlled trial, stroke

## Abstract

**Introduction:**

Post-stroke cognitive impairment (PSCI), which has a high morbidity, is closely associated with the recurrence and rehabilitation of ischemic stroke. There are 2 different stages of PSCI, including post-stroke cognitive impairment with no dementia (PSCIND) and post-stroke dementia (PSD). The latter has a significantly higher mortality rate than the previous one. Therefore, preventing the onset of PSD is of vital importance. However, there is no unequivocally effective prevention or treatment for PSCI, except intensive secondary prevention of stroke. The primary aim of this protocol is to explore whether acupuncture can improve cognitive function of patients with PSCIND and reduce the chances of developing PSD. On this bias, we also want to explore its possible mechanisms.

**Methods and analysis:**

A prospective, multicenter, large sample, randomized controlled trial will be conducted. A total of 360 eligible patients will be recruited from 5 different hospitals and randomly allocated into the acupuncture group (AG), sham acupuncture group (NAG), and waiting-list group (WLG) in a 1:1:1 ratio. The intervention period of NAG and AG will last 3 months (30 minutes per day, 3 times per week). Primary and secondary outcomes will be measured at baseline, 12 weeks (at the end of the intervention), 24 weeks (after the 12-week follow-up period), and 36 weeks (after the 24-week follow-up period). Resting-state and task-state functional MRI will be conducted at baseline and 12 weeks.

**Ethics and dissemination:**

The ethic committee of First Teaching Hospital of University of Traditional Chinese Medicine approved the study. Study results will be first informed to each participant and later disseminated to researchers, and the general public through courses, presentations and the internet, regardless of the magnitude or direction of effect. The results will also be documented in a published peer-reviewed academic journal.

**Registration:**

We have registered at ClinicalTrials.gov(ChiCTR2000033801).

## 1 Introduction

Stroke is one of the leading causes of death and disability in the world.^[1,2]^ A study^[3]^ shows that even minor stroke can influence activities of daily living (ADLs), cognitive function, and quality of life. This brought great financial burden to patients and society.^[4,5]^ Stroke survivors are at an increased risk of developing cognitive impairment.^[6]^ Post-stroke cognitive impairment (PSCI) has a high morbidity; a recent follow-up study^[7]^ shows that prevalence of PSCI was 61% among 10-year stroke survivors. PSCI is also associated with recurrence of ischemic stroke in high-risk patients during adequate medical therapy including antiplatelet therapy. The lowest tertile of Mini-mental State Examination (MMSE) score (0∼23) was independently associated with the risk of recurrent ischemic stroke.^[8]^

PSCI contains 2 different degrees of cognitive impairment, including post-stroke cognitive impairment with no dementia (PSCIND) and post-stroke dementia (PSD). In America, a study on 212 subjects from the Framingham Study suggested that 19.3% of cases developed into the dementia in 10 years after stroke.^[9]^ In order to prevent the onset of dementia, early diagnosis and intervention of PSCIND is vitally important. The AHA/ASA Guidelines for adult stroke rehabilitation and recovery (2016) recommends that all clinical stroke patients should get screening of cognitive status before leaving hospital.^[10]^ However, there is no unequivocally effective treatment for PSCI patience. So far, the best way to prevent PSD is to prevent stroke recurrence and severity by using optimal acute treatment and intensive secondary prevention.^[11]^ Cholinesterase inhibitors commonly used in Alzheimer disease (AD), such as donepezil, also have positive effects on PSCI patience.^[12–14]^ However, the uncertainty on the global and daily function makes it difficult to evaluate the worth of the drugs on clinic.^[15]^ Therefore, it is of vital importance to explore effectively treatment to slow or stop disease progression. In recent years, studies^[16]^ have shown that acupuncture combined medication, such as donepezil or nimodipine, is more beneficial to cognitive function of PSCI patience. But there are few studies that focus on the individual effects of acupuncture on PSCI.

Therefore, the primary aim of this study is to explore whether acupuncture can improve cognitive function of patients with PSCIND and reduce the chances of developing PSD. Furthermore, we also want to explore its possible mechanisms. Recent studies show that PSCI is a result of mixed damage mechanisms, but the ongoing ischemic vascular processes may be the main mechanism.^[17]^ However, there is still a lack of studies on the mechanism of acupuncture on improving cognitive function. Therefore, in this parallel study, we want to not only probe into the effect of acupuncture, but also explore its mechanism by using function-MRI and analysis of amyloid beta (Aβ).

## 2 Methods and analysis

### 2.1 Objective

The aim of this study was to evaluate the efficacy of acupuncture treatment on improving cognitive function, ADLs, mental state, and quality of life for PSCIND patients, and also explore its possible mechanisms.

### 2.2 Trial design

**Figure 1:**
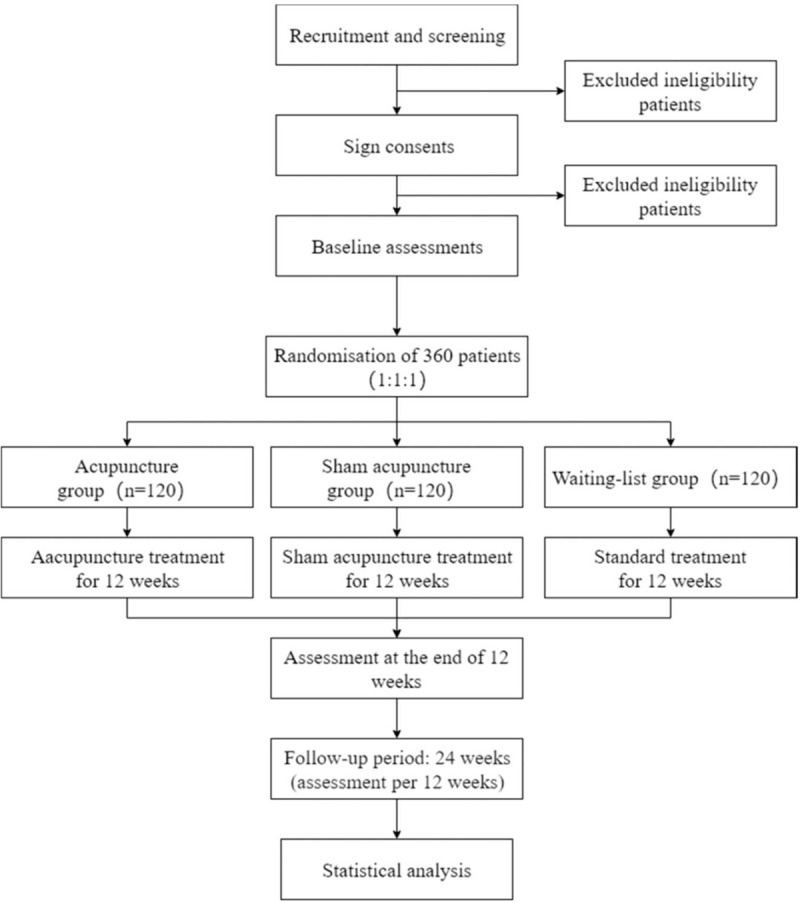
Flowchart of the trial design.

This is a multicenter, single-blind, randomized controlled trail that participants will be random allocated to 3 groups with a 1:1:1 allocation ratio. The 3 groups are acupuncture group (AG), sham acupuncture group (NAG), and waiting-list group (WLG). The flowchart of the trial design is shown in Figure 1.

### 2.3 Inclusion criteria

Patients are eligible for study inclusion if they

(1) Meet the diagnostic criteria of ischemic stroke^[18]^
(2) Meet the diagnostic criteria of PSCIND^[7,19–21]^

(1) Have the ability to take care of themselves in daily life. Barthel index ≥60.
(2) Montreal cognitive assessment scale (MoCA) < 26
(3) Mini-mental State Examination (MMSE): MMSE Score >17 (illiteracy), or MMSE score > 20 (primary school culture), or MMSE score >24 (middle school and above);
(4) Ischemic stroke within 6 months;
(5) Men or women aged 18 to 75 years;
(6) The patients sign informed consent and cooperate with the treatment.

### 2.4 Exclusion criteria

Patients will be excluded if they have any one of the following:

(1) Patients were diagnosed as having cognitive impairment before cerebral infarction;
(2) Patients were taking medications that may interfere in the efficacy assessment of the study therapies, including cholinesterase inhibitors, cytosolic sodium, or receiving acupuncture to treat cognitive impairment;
(3) Patients who are unable to complete assessments during the screening process due to serious mental, cognitive, or emotional disorders;
(4) Patients who have severe diseases in the internal organs, including the heart, liver, and kidney;
(5) Patients live alone without long-term living partners or caregiver;
(6) Women who are pregnant or preparing to pregnant or breast-feeding;
(7) Patients who are recruited into other clinical trials which related to acupuncture or cognitive impairment within 1 month.

### 2.5 Endpoint event

The patient meets the diagnostic criteria for dementia: MMSE score ≤17 (illiteracy), or MMSE score ≤ 20 (primary school culture), or MMSE score ≤24 (middle school and above).

### 2.6 Withdrawal or dropout criteria

Participants will be terminated to continue this trial according to the following criteria:

(1) The participants who have a deteriorated condition or have had a serious adverse event;
(2) The participants who suffer from certain complications or specific physiological changes during the study may not be appropriate to continue;
(3) The participants who are unwilling to continue the study.

### 2.7 Recruitment strategies and randomization

The study population is patients who had Post-stroke Cognitive Impairment with no dementia. We will recruit 360 eligible patients from five hospitals in China: The First Teaching Hospital of Tianjin University of traditional Chinese medicine (TCM), The Changchun hospital of TCM, Changzhi City of Traditional Chinese Medicine affiliated hospital, Changsha central hospital, The Second affiliated hospital of Baotou. Recruitment posters will be used in every participating hospital. We also use the various social media platforms to distribute the trial information. The eligible patients must satisfy the criteria defined below. Patients who express a general interest in taking part in the study will be informed of all necessary information in oral and written form. After fulfilling the criteria and signing the consent, the study personnel will assign the patient to the acupuncturist who is in charge of the patient.

A full-time staff of The First Teaching Hospital of Tianjin University of TCM will randomize all the eligible participants into the AG, NAG, or the WLG in a 1:1:1 ratio by a dynamic random allocation technique.

### 2.8 Blinding

Assessors who are masked to treatment allocation and trained will be in charge of baseline evaluation and measurement. Patients who receive acupuncture treatment will not be aware of their group assignment. However, the waiting-list group cannot be blinded. Because different acupuncture treatments are used in AG and NAG, it is impossible to blind the acupuncturists. During the trial period and follow-up phases, the third party and assessors will all be blinded.

## 3 Interventions

All the groups receive standard treatment that contains controlling blood pressure, blood glucose, blood lipid, symptomatic treatment.

### 3.1 Acupuncture group (AG)

Patients in AG will be given TiaoShenYiZhi acupuncture therapy.^[22]^ To ensure the optimal effects of acupuncture stimulation, all the therapists are required to have clinical experience of acupuncture treatments for at least 5 years.

Participants will be asked to adopt sitting or supine position according to their condition. Disposable, sterile needles with a diameter of 0.25 mm and a body length of 40 mm (HUATO, Suzhou, China) will be used. A total of 12 acupoints were selected in the acupuncture therapy, including Neiguan (PC6), Renzhong (DU26), Baihui (DU20), Sishencong (EX-HN1), Fengchi (GB20), Wangu (GB12), Tianzhu (BL10), Sibai (ST2), Shenmen (HT7), Fenglong (ST40), Sanyinjiao (SP6), and Taichong (LR3). PC6, GB20, GB12, BL10, ST2, HT7, ST40, SP6 are used bilaterally. The location and manipulations of the aforementioned acupoints are listed in Table 1. All the manipulations will be applied for 1 minute and the needles are retained for 30 minutes. The participants will be provided acupuncture treatments 3 times per week for 3 months.

**Table 1:** Acupoints selected for use in the study.

Acupoints	Location	Manipulation
Neiguan (PC6)	On the forearm, 2 cun above the transverse crease of the wrist, between the tendons of m. palmaris longus and m. flexor radialis	Vertical insertion with a depth of 0.5--1 cun is applied with the twisting, lifting and thrusting technique of the reducing method for 1 minute
Renzhong (DU26)	On the face, at the junction of the upper third and middle third of the philtrum	Oblique insertion with 0.3--0.5 cun in depth toward the nasal septum and heavy bird-pecking needling is applied until tear formation was observed in patients’ eyes.
Baihui (DU20)	On the midline of the head, 5 cun directly above the midpoint of the anterior hairline, approximately on the midpoint of the line connecting the apexes of both ears	Horizontal insertion with 1 cun in depth is applied with small amplitude and high frequency twisting technique of the reinforcing method for 1 min
Sishencong (EX-HN1)	On the head, a group of 4 points, at the vertex, 1 cun respectively posterior, anterior and lateral to BaiHui (DU20)	
Fengchi (GB20)	On the nape, below the occipital, on a level with Fengfu (DU-16), in the depression between the upper portion of trapezius and the sternocleidomastoid	Vertical insertion with a 1--1.5 cun in depth is applied with small amplitude and high frequency twisting technique of the reinforcing method for 1 minute
Wangu (GB12)	On the nape, in the depression posterior and inferior to the mastoid process	
Tianzhu (BL10)	On the nape, 1.3 cun lateral to the mid-point of the posterior hairline and in the depression on the lateral aspect of m. trapezius	
Sibai (ST2)	On the face, directly below the pupil, in the depression at the infraorbital foramen	Vertical insertion with a 0.3--0.5 cun in depth is applied with mild reinforcing-reducing method for 1 min
Shenmen (HT7)	On the wrist, at the ulnar end of the transverse crease of the wrist, in the depression on the redial side of the tendon of m. flexor carpi ulnaris	
Fenglong (ST40)	On the crus, 8 cun superior to the tip of the external malleolus, lateral to Tiaokou (ST38) about 2 finger-breadth lateral to the anterior border of the tibia	Vertical insertion with a depth of 1.0–1.5 cun is applied with the twisting, lifting, and thrusting technique of the reducing method for 1 min
Taichong (LR3)	On the dorsum of the foot, in the depression distal to the junction of the first and second metatarsal bones	
Sanyinjiao (SP6)	On the crus, 3 cun directly above the tip of the medial malleolus, posterior to the medial border of the tibia	Vertical insertion with a depth of 1.0–1.5 cun is applied with the twisting technique of the reinforcing method for 1 min

### 3.2 Sham acupuncture group (NAG)

**Figure 2:**
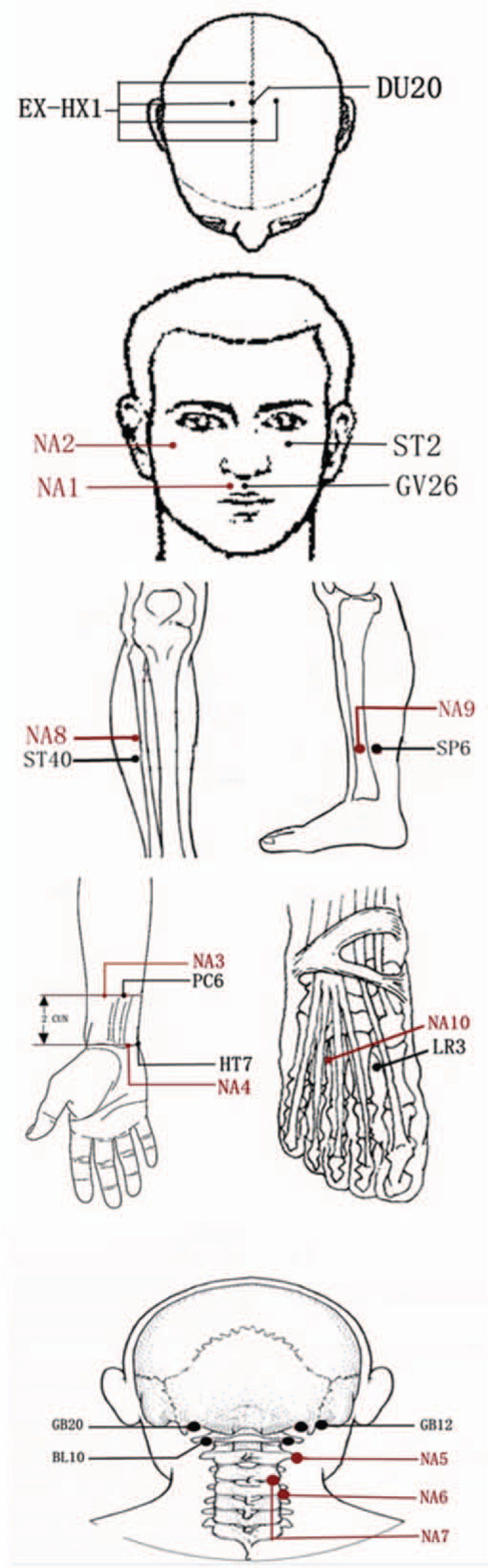
Location of the acupoints and non-acupoints used in the study.

Acupuncture will be performed at 10 non-acupoints. The selection of non-acupoints in NAG are the points locating on non-meridians, but near the acupoints we select in AG, which is a common method in the design of sham acupuncture.^[23]^ Shallow needling method will be used on these non-acupoints, without making sensation of Deqi. The protocol for choosing the non-acupoints are listed and shown in Table 2 and Figure 2. The requirements for acupuncturists are the same as for AG.

**Table 2:** The location of non-acupoints.

Non-acupoint	Location	Manipulation
Non-acupoint 1 (lei Renzhong)	On the face, directly below the right nostril, 0.5 cun next to Renzhong (DU26)	Prick straight and lightly, make the needles hang on the skin
Non-acupoint 2 (lei Sibai)	On the face, directly below the Outside canthus, on a level with Sibai (ST2), in the sutura between upper jaw bone and zygomatic bone	
Non-acupoint 3 (lei Neiguan)	On the palmar side of the forearm, on a level with Neiguan (PC6) and 1 cun radialis to it (between the Pericardium Meridian of Hand-Jueyin and the Lung Meridian of Hand-Taiyin).	
Non-acupoint 4 (lei Shenmen)	On the wrist, at the transverse crease of the wrist, at the midpoint of Shenmen (HT7) and Daling (PC7) (between the Pericardium Meridian of Hand-Jueyin and the Heart Meridian of Hand-Shaoyin).	
Non-acupoint 5 (lei Fengchi)	On the nap, 1 cun below the outside of Tianzhu (BL10)	
Non-acupoint 6 (lei Wangu)	On the nap, 2 cun lateral to posterior median line, at the level of the lower border of the spinous process of the 4th cervical vertebra	
Non-acupoint 7 (lei Tianzhu)	On the nap, 1 cun lateral to posterior median line, at the level of the lower border of the spinous process of the 3rd cervical vertebra	
Non-acupoint 8 (lei Fenglong)	On the crus, 6 cun below Dubi (ST 35), lateral to Shangjuxu (ST37) and two finger-breadth from the anterior border of the tibia	
Non-acupoint 9 (lei Sanyinjiao)	On the crus, at the midpoint of the medial side of the tibia, on a level with Sanyinjiao (SP6)	
Non-acupoint 10 (lei Taichong)	On the dorsum of the foot, in the depression distal to the junction of the third and fourth metatarsal bones, at the level of Taichong (LR3)	

### 3.3 Waiting-list group

No intervention except standard treatment will be performed in the waiting-list control group in the initial 36 weeks after randomization.

### 3.4 Follow-up

After the 12-week intervention period, all participants will enter an additional 24-week follow-up period. Participants will be followed up per 12 weeks since the treatment end. Assessors will contact with the participants by telephone before the follow-up date. If there is no response to the telephone contact for 1 consecutive week, or the patient expresses that he/she does not want to cooperate with the follow-up, he/she will be listed as lost, the follow-up work should be stopped.

Participants will be given acupuncture treatment when the follow-up period is completed, in order to reduce the shedding rate.

### 3.5 Outcome assessment

In this trail, most primary and secondary outcomes will be measured at baseline, the end of intervention(12weeks), after an additional 12-week follow-up period (24 weeks) and after an additional 24-week follow-up period (36 weeks). Amyloid-β and fMRI will be measured at baseline and the end of intervention. A summary of all the measures in the trial is summarized in Table 3.

**Table 3:** Outcome measurements at each timepoint.

Study period	Enrolment	Intervention	Follow-up	
STUDY TIMES	The first time	The second time	The third time	The fourth time
TIME POINT	−3d-0	12 wks ± 3d	24 wks ± 3 d	36 wks ± 3d
MoCA	×	×	×	×
MMSE	×	×	×	×
CDR	×	×	×	×
NPI	×	×	×	×
WHOQOL-BREF	×	×	×	×
FAQ	×	×	×	×
β-Amyloid	×	×		
Adverse Reaction/Event		×	×	×
Vital signs	×	×	×	×
Recurrence		×	×	×
Using drugs	×	×	×	×
Laboratory examination	×	×		

### 3.6 Primary outcome

The primary outcome of this study is the cognitive function and incidence of PSD.

### 3.7 Cognitive function

For screening cognitive decline, MMSE is the most common used screening scale.^[24]^ It contains domains of orientation, memory, attention, language, and visuospatial ability.^[25]^ However, studies also showed that MMSE has deficiency on detecting mild cognitive impairment (MCI) and clinical signs of dementia.^[26,27]^ It is superior for more advanced stages, meanwhile MoCA is more useful for the mild stages of the cognitive impairment.^[28]^

Thus, we also use MoCA, which is a cognitive screening, diagnostic, and tracking tool with high sensitivity and specificity for detecting MCI,^[28]^ to evaluate the cognitive function. MoCA also has superiority on detecting executive dysfunction compared with the MMSE.^[29]^ It assesses several cognitive domains such as memory, executive function, attention, language, abstraction, naming, delayed recalls, and orientation.^[30]^ In order to avoid false increase of participants’ cognitive-function test results caused by repeated tests, we will use different versions of MoCA, including 7.1, 7.2, 7.3, and basic of MoCA.

We also choose Clinical Dementia Rating Scale (CDR) to evaluate cognitive function. A CDR of 0.5 is established risk states for dementia.^[31]^ Compared with MMSE, although CDR score is not predictive of change in cognitive performance, it is predictive of functional change over time and of conversion to dementia.^[32]^

### 3.8 Incidence of PSD

We will use MMSE, which is the most widely used by frontline physicians, to detecting dementia. Dementia: MMSE score ≤17 (illiteracy), or MMSE score ≤20 (primary school culture), or MMSE score ≤24 (middle school and above).^[33]^

## 4 Secondary outcome

### 4.1 Instrumental activities of daily living (IADL)

Compared with basic ADLs, functional changes are noted earlier in the dementia process with instrumental activities of daily living (IADLs).^[34]^ We will use the Functional Activities Questionnaire (FAQ), which has a wide application to measure the IADLs. The target population of FAQ includes adults with normal cognition, MCI, as well as mild, moderate, and advanced dementia.^[35]^

### 4.2 Neuropsychiatric inventory (NPI)

The evaluation of neuropsychiatric symptoms is an important part of dementia diagnosis and management.^[36]^ The 12-item version of neuropsychiatric inventory (NPI) will be used in this trial. Compared with the original NPI with 10 items, this version added the elements of sleep/nighttime, behavior disturbances, and appetite/eating disturbances.^[37]^

### 4.3 Quality of life (QOL)

The cognitive decline has a great influence on the quality of patience's life. In this trail, QOL will be evaluated by WHOQOL-BREF Chinese Scale.

### 4.4 Amyloid-β

Previous studies^[38]^ showed that stroke can enhance the vascular deposition of amyloid-β (Aβ), which induced cognitive decline and post-stroke cognitive impairment. For the 2 main forms of Aβ, the 40-amino acid form (Aβ 40) and the minor 42-amino acid form (Aβ 42), the aggregates of Aβ42 is neurotoxic and is more readily than Aβ 40.^[39]^ So, this trial choose Aβ 42 as the laboratory index.

The patients’ blood samples will be collected and analyzed twice, one before the treatment, another after 3 months treatment. The blood sample (5 mL) from each patient will be taken under aseptic conditions from the antecubital vein and will be placed in EDTA anticoagulant tubes. After centrifugation at 3000 r/min for 10 minutes, the extracted serum will be put into tubes and be stored at −80°C until the analysis. The level of plasma Aβ42 will be detected by the enzyme-linked immunosorbent assay (ELISA).

## 5 Data management and quality control

The third party is fully responsible for the data management of the project. Specific research assistants (RA), who are masked to treatment allocation, will double-enter the data electronically. Data will be collected by medical records, asking patients, participants’ relatives, caregivers, or proxies directly. The personal information of the participants will be kept in specific files with pre-set codes. Entering data into eCRFs and other essential skills were covered during the training session before study commencement.

We will set up a quality control group to guarantee the validity and reliability of results. Every 3 months, members of the quality control group will perform a quality control review at each hospital and produce a report regarding the quality analysis of the research process.

## 6 Safety evaluation

Subcutaneous hemorrhage, hematoma, syncope, stuck-needle are the common adverse events (AE) of acupuncture treatment. We will record all the adverse events of acupuncture during the intervention and follow-up period. The proportion, type, and potential reasons of AEs will be calculated. All AEs will get appropriate intervention and serious AEs will be reported to the ethics committee immediately.

## 7 fMRI acquisition

### 7.1 Objective

In terms of brain network and brain functional connections, this trial also aims to explore the mechanism of acupuncture treatment for cognitive impairment after stroke.

### 7.2 Participants

Twenty eligible participants will be screened in each group (AG, NAG, WLG).

### 7.3 Method

Resting-State fMRI Acquisition.

### 7.4 Assessment

For AG and NAG, fMRI data will be collected at enrollment and after the last treatment. For WLG, fMRI data will be collected once at enrollment and after 3 months.

### 7.5 Sample size and statistical methods

The sample size calculation is based on the improvement of Montreal cognitive assessment scale (MoCA) scores, which has a higher sensitivity than MMSE for MCI.^[7]^ According to the similar published articles,^[40]^ the mean difference in MoCA scores between AG and control group (CG) is 1.5, with a standard deviation of 1.13 in AG and 1.20 in CG. According to our previous study, acupuncture treatment can probably improve the MoCA by 0.6 on average compared with control group. Considering a 1-sided significance level of 0.025 and power of 95%, 95 participants are required for each group, as calculated by PASS 11 software. To minimize attrition bias, we assumed the dropout rate of 20%, making it necessary to include at least 357 participants with a 1:1:1 group allocation rate. We plan to enroll 360 participants in total.

Continuous variables are presented as mean (SD) if they obey normal distribution, and paired-sample *t* test will be used in intragroup comparison; if they are not normal distribution, median (quartile interval) [M(IQR)] will present them, and Wilcoxon rank sum test will be used in intra-group comparison; Variance analysis will be used for comparison between groups which is consistent with the normal distribution and homogeneity of variance; if not, then Mann–Whitney *U* test will be used. Group comparisons will then be undertaken using χ^2^ tests for categorical characteristics. Two-sided *P* < .05 represents statistical significance for all the analyses.

## 8 Discussion

By reason of the high prevalence of PSCIND and high social acceptability of acupuncture in China, we have the advantage on conducting large-sample research. This protocol design also has additional strengths. First, as far as we know, we are the first randomized controlled trial to study if acupuncture has efficacy on preventing PSCIND progressing to PSD; second, avoiding false increase of patients’ cognitive-function test results caused by multiple use of same scale. We measure the cognitive function with different version of MoCA, including 7.1, 7.2, 7.3, and basic of MoCA; third, multicenter. Our trial will be conducted in 5 inpatient and outpatient stroke rehabilitation units of different cities, which can ensure the sample representativeness. Fourth, a 12-week intervention and 24-week follow-up assessment can provide reliable long-term effect evidence for acupuncture on improving cognitive function, mental state, and quality of life for PSCIND patients.

On the contrary, this protocol design also has limitations, which are as follows. There could be a very few overlaps exists between early stage of AD and PSCIND in our participants. Fifty-six percent of all demented cases are the patients who suffer from AD with stroke.^[41]^ A Study also shows that the atherosclerosis, which is one of the common causes of stroke, may also have effects on the pathogenesis of AD.^[42]^ We can see that the inner correlation may exist between stroke and AD. Therefore, the participants in our trail may also suffer both AD and PSCI. And this will affect the evaluation of the outcome in this study. Potential spontaneous recovery at the early stage of stroke may have the confounding effects on this trial, but the waiting-list group can reduce the effects of it. Due to the limitations of equipment and capital, only part of the participants will get the fMRI scan. This may affect the generalizability of the results.

## 9 Ethics approval and consent to participate

The ethic committee of First Teaching Hospital of University of Traditional Chinese Medicine approved the study. We have also registered at ClinicalTrials.gov (http://www.chictr.org.cn/showprojen.aspx?proj=54764): ChiCTR2000033801. All patients gave their written informed consent before participating in the clinical trial. The details of our study, including trial objectives, characteristics, probable benefits and risks, other available treatment alternatives, and the subjects’ rights as well as obligations as stated in the Declaration of Helsinki, will be made clear to the patients by a study personnel who has their grouping code, and their agreement will be required. After obtaining the written informed consent, the subjects will be enrolled in the study. During the process of the trial, if new points regarding the study ethics emerge, written revisions regarding the informed consent will be handled by the Ethics Committee and, after their approval, the individuals’ consent will be requested again. In case of the patients’ withdrawal, their available data will be kept for the final analyses.

## 10 Dissemination

Study results will be first informed to each participant and later disseminated to researchers, and the general public through courses, presentations and the internet, regardless of the magnitude or direction of effect. The results will also be documented in a published peer-reviewed academic journal.

### 10.1 Consent for publication

Not applicable.

### 10.2 Availability of data and materials

The submitted manuscript is a study protocol that includes no primary data now. More data and materials unmentioned can be obtained from the corresponding author by the contact methods provided in the manuscript.

### 10.3 Patient and public involvement

Patients and the public were not involved in in the design, or conduct, or reporting, or dissemination plans of our research.

### 10.4 Informed consent statement

All patients will be provided informed consent for publication of the case.

## Author contributions

YZD conceived the study design and provided the professional advice on the drafting profile. LLZ, WL, and CR drafted the manuscript. BXL and XN revised the paper and drew the tables and the figure. HLJ and ZFL polished the final manuscript. All authors read and approved the final manuscript.

**Supervision:** Xi Nan, Zefang Li, Hailun Jiang.

**Writing – original draft:** Lili Zhang, Wei Liu, Chang Rao, Boxuan Li.

**Writing – review & editing:** Yu-zheng Du, Xi Nan, Zefang Li, Hailun Jiang.
